# MicroRNA profiling of cisplatin-resistant oral squamous cell carcinoma cell lines enriched with cancer-stem-cell-like and epithelial-mesenchymal transition-type features

**DOI:** 10.1038/srep23932

**Published:** 2016-04-05

**Authors:** Ruma Dey Ghosh, Sangeeta Ghuwalewala, Pijush Das, Sapan Mandloi, Sk Kayum Alam, Jayanta Chakraborty, Sajal Sarkar, Saikat Chakrabarti, Chinmoy Kumar Panda, Susanta Roychoudhury

**Affiliations:** 1 Cancer Biology and Inflammatory Disorder Division, CSIR-Indian Institute of Chemical Biology, Kolkata, India; 2 Structural Biology and Bio-Informatics Division, CSIR-Indian Institute of Chemical Biology, Kolkata, India; 3 Department of Surgical Oncology, Chittaranjan National Cancer Institute, 37, S.P. Mukherjee Road, Kolkata, India; 4 Department of Oncogene Regulation, Chittaranjan National Cancer Institute, 37, S.P. Mukherjee Road, Kolkata, India

## Abstract

Oral cancer is of major public health problem in India. Current investigation was aimed to identify the specific deregulated miRNAs which are responsible for development of resistance phenotype through regulating their resistance related target gene expression in oral squamous cell carcinoma (OSCC). Cisplatin-resistant OSCC cell lines were developed from their parental human OSCC cell lines and subsequently characterised. The resistant cells exhibited enhanced proliferative, clonogenic capacity with significant up-regulation of P-glycoprotein (ABCB1), c-Myc, survivin, β-catenin and a putative cancer-stem-like signature with increased expression of CD44, whereas the loss of E-cadherin signifies induced EMT phenotype. A comparative analysis of miRNA expression profiling in parental and cisplatin-resistant OSCC cell lines for a selected sets (deregulated miRNAs in head and neck cancer) revealed resistance specific signature. Moreover, we observed similar expression pattern for these resistance specific signature miRNAs in neoadjuvant chemotherapy treated and recurrent tumours compared to those with newly diagnosed primary tumours in patients with OSCC. All these results revealed that these miRNAs play an important role in the development of cisplatin-resistance mainly through modulating cancer stem-cell-like and EMT-type properties in OSCC.

Head and neck squamous cell carcinoma (HNSCC) is the sixth most common cancer worldwide^1^. It is one of the most prevalent and leading cancers in India^2^. It accounts for over 30% of all cancers reported in the country^3^. In head and neck cancers, oral squamous cell carcinoma (OSCC) arises from the epithelial lining of the oral cavity, pharynx, larynx and it accounts 90–94% of all oral cancers^4^. Although, smoking is a primary risk factor for oral cancers, the other etiological factors include the use of alcohol, areca nut, betel leaf in addition to human papillomavirus (HPV) infection^2^,^3^. The treatment of OSCC involves surgery, radiotherapy and chemotherapy. Despite advancement in both diagnosis and therapy in recent years, the prognosis and 5-year survival rate of OSCC remains same at around 50%^5^. Fatal outcome is mainly caused by local recurrence and cervical (neck) lymph node metastasis and occasionally by distant organ metastasis. Nonetheless, due to their heterogeneous character, it is difficult to distinguish good prognostic tumour from more-aggressive poor prognostic tumour which shows therapy resistance and subsequently relapses and metastasizes^5^. In this patient subgroup (poor prognostic) the selection of pre-existing tumourogenic resistant cells or the cancer-stem-cells (CSCs) and/or acquisition of resistant cells during treatment with chemo-radiation therapy has been anticipated^6^. The drug-resistance is mediated either with the over-expression of multidrug resistance (MDR) related ABC-transporters, growth factor receptors, or through acquisition of cancer stem-cell-like (CSC), epithelial-mesenchymal transition (EMT) properties and activation of DNA-repair mechanism^7^,^8^,^9^,^10^. Subsequently, deregulated miRNAs play an important role in the regulation of tumour recurrence and metastasis^11^,^12^. Moreover, the exposures to environmental toxic agents (smoking) are able to alter miRNA expression and thus implicating them in cancer development^13^. However, the involvement of miRNAs in the development of drug-resistance in OSCC has not been understood clearly.

In the present study we have developed two cisplatin-resistant OSCC cell lines that provided an insight into the drug resistant phenotype in oral cancer. We found together with the activation of the drug resistance the enrichment of cancer stem-cell-like property coupled with augmentation of EMT phenotype in OSCC cell lines. We further identified a miRNA expression signature associated with the drug resistance property of these cell lines. Consequently, we have checked the expression level of these miRNAs in OSCC-patient sub-groups like primary tumour, neoadjuvant chemotherapy treated tumour and recurrent tumour. In this study, we aim to understand the molecular pathways by which enrichment of cancer-stem-cell markers associated with induced EMT occurs in these cell lines anticipating that it might have a role in therapeutic outcome, metastasis and tumour recurrence.

## Results

### Cisplatin-resistant cells confer resistance to cisplatin-induced-cytotoxicity

**Figure 1:**
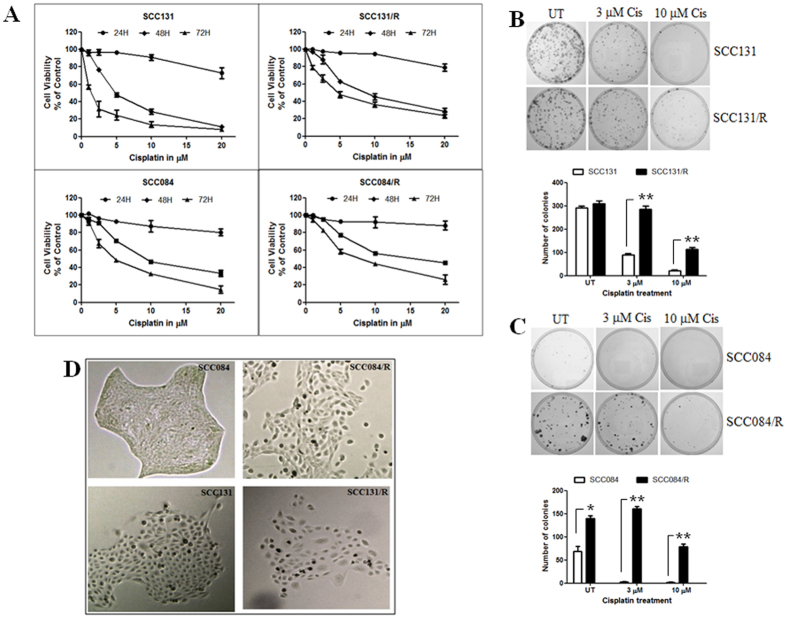
Cytotoxic effect of cisplatin on cisplatin-resistant (SCC131/R and SCC084/R) and their parental (SCC131 and SCC084) OSCC cells lines. (**A**) Dose response curves of cell viability for cisplatin using SCC131, SCC131/R (upper panel), and SCC084, SCC084/R (lower panel) cells for different time point (24 h: •, 48 h: ♦, 72 h: ▲) as assessed by MTT assay. Cell viability was determined as percentage of untreated control cells. Value represents the mean ± SE of three independent experiments with four replicates in each. (**B,C**) Cytotoxicity of cisplatin in resistant and parental SCC131 (**B**) and SCC084 (**C**) cells was also determined by monolayer colony formation assay. Clonogenicity of cells was affected by different doses of cisplatin as seen in the plates (upper panel). The colonies were counted from three independent experiments using Image J software and plotted as mean ± SE (lower panel). *p < 0.05, **p < 0.01, ***p < 0.001 (**D**) Untreated parental and resistant cell colonies (upper panel for SCC084 and lower panel for SCC131) were evaluated and found with distinct morphological differences using light microscope (10X magnification). Resistant cells were composed mainly of spindle-shaped cells along with heterogeneous population compared to the parental cells.

Preliminary information on two oral squamous cell carcinoma cell lines UPCI: SCC084 and UPCI: SCC131 (hereafter referred to as SCC084 and SCC131) is given in the Supplementary Table S1^14^. Cisplatin resistant cell lines, SCC084/R and SCC131/R were developed by incremental doses of cisplatin treatment in successive passages for 6 months (see material and method section) from their parental cell line, SCC084 and SCC131 and maintained in presence of 3 μM cisplatin. Cell lines were exposed to various concentrations (1 μM to 20 μM) of cisplatin for different time points (24 h, 48 h, and 72 h) to determine the cisplatin-induced-cytotoxicity. The cell viability profile of the cisplatin-resistant (SCC084/R and SCC131/R) and their parental (SCC084 and SCC131) cell lines are shown in Fig. 1A. The IC_50_ value of SCC084/R and SCC131/R cells were increased by 1.6 and 3.5 fold (R index) upon 72 h incubation with cisplatin respectively as compared to their parental cells corroborating the fact that the number of viable cells is significantly high in cisplatin-resistant cells (Fig. 1A, Supplementary Table S2). Moreover, the results also revealed that the parental SCC084 cell itself, originated from a recurrent tumour, was more resistant to cisplatin and it showed approximately 3.5 fold higher (at 72 h) IC_50_ value compared to the SCC131 cell line which was originated form a primary tumour (Supplementary Table S2).

We also determine the cisplatin-induced-cytotoxicity of these OSCC cell lines by monolayer colony formation assay at two different doses of cisplatin: 3 μM (in which the resistant cell lines were maintained) and 10 μM (a much higher dose than the IC_50_ values of cisplatin-resistant cells at 72 h). The results showed that pre-treatment with cisplatin for 24 h significantly increased the colony formation efficiency and number of colonies after 14 days in cisplatin-resistant cells than those of their parent cells (Fig. 1B,C). Interestingly we observed that cisplatin-resistant and sensitive cells have distinct cellular morphology. The compactness of the colonies was reduced and loosely dispersed in case of resistant cells. The shape and size were also significantly changed in cisplatin-resistant cells compare to parent cells. The morphologically heterogeneous population could also be frequently recognised in resistant cell-colonies especially in SCC131/R cell colonies (Fig. 1D).

### Cisplatin-induced apoptosis is significantly abrogated in the resistant OSCC cells

**Figure 2:**
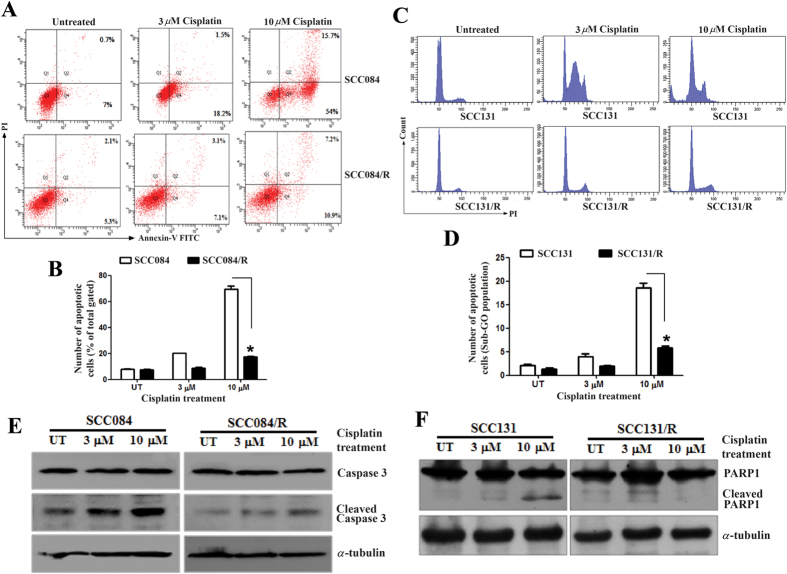
Cisplatin induces apoptosis in parental cells significantly compare to resistant OSCC cells. (**A**) Quantification of apoptosis by annexin V binding to SCC084 and SCC084/R cells. Cells were incubated with indicated doses of cisplatin for 48 h and then stained with annexin V-FITC/PI and analyzed by flow cytometry. (**B**) Bar diagram represent the mean ± SE calculated from three independent experiments. (**C**) Cell cycle distribution of SCC131 and SCC131/R cells following cisplatin treatment for 48 h. After staining with propidium iodide they were analyzed using a flow cytometer. (**D**) Each column represents the percentages of cells in sub-dipliodal (Sub-G_0_/G_1_) population of cell cycle at indicated time after treatment. Data represent the mean ± SE calculated from three independent experiments. (**E**) Representative image of caspase 3 cleavage by western blot using cleaved Caspase 3 antibody in SCC084 and SCC084/R cells. Cells were incubated with indicated doses of cisplatin for 48 h and then whole cell lysates analyzed by western blot for caspase 3 and cleaved caspase 3 protein product. α-Tubulin was used as a loading control. (**F**) Effect of cisplatin on the cleavage pattern of PARP1 in SCC131 and SCC131/R cells. Cells were incubated with indicated doses of cisplatin for 48 h and then whole cell lysates were for western blotting for cleaved and total PARP1 using specific antibody. α-Tubulin was used for normalization. For **(A,C,E)** and **(F)**, images are representative of three independent experiments. *p < 0.05, **p < 0.01, ***p < 0.001.

Next we performed the following experiments to elucidate cisplatin-resistant OSCC cell lines exhibit attenuated apoptosis upon drug treatment. The FACS analysis of annexin V-FITC and propidium iodide (PI) stained cells showed that cisplatin mediated apoptotic cell (both annexin V-FITC positive and annexin V-FITC/PI positive) population was significantly high in parental, SCC084 and SCC131 cells compared to the resistant, SCC084/R and SCC131/R cells (Fig. 2A,B). FACS analysis was also employed to determine the effect of cisplatin on the cell cycle phase distribution of both resistant and sensitive cell lines. The appearance of sub-diploid phase (sub G1/G0) is a specific marker of apoptosis. The significant increase in the counts of sub diploidal cells in a dose dependent fashion were found only in sensitive SCC084 and SCC131 cells compared to the untreated control and the resistant, SCC084/R and SCC131/R cells (Fig. 2C,D). Further to prove the differential response of OSCC resistant cells to cisplatin, we evaluated the caspase-3 and PARP1 cleavage in these cell lines by western blot analysis (Fig. 2E,F). Our data revealed that both caspase-3 and PARP1 cleavages were distinct after treatment of cisplatin for 72 h in SCC084 and SCC131 cells compared to that of resistant cells SCC084/R and SCC131/R (Fig. 2E: representative data for Caspase-3 cleavage in SCC084 cell line and Fig. 2F: representative data for PARP1 cleavage in SCC131 cell line). Collectively, these observations revealed that SCC084/R and SCC131/R cells confer resistance by abrogating the cisplatin-induced apoptosis.

### Molecular characterisation of cisplatin-resistant cells

**Figure 3:**
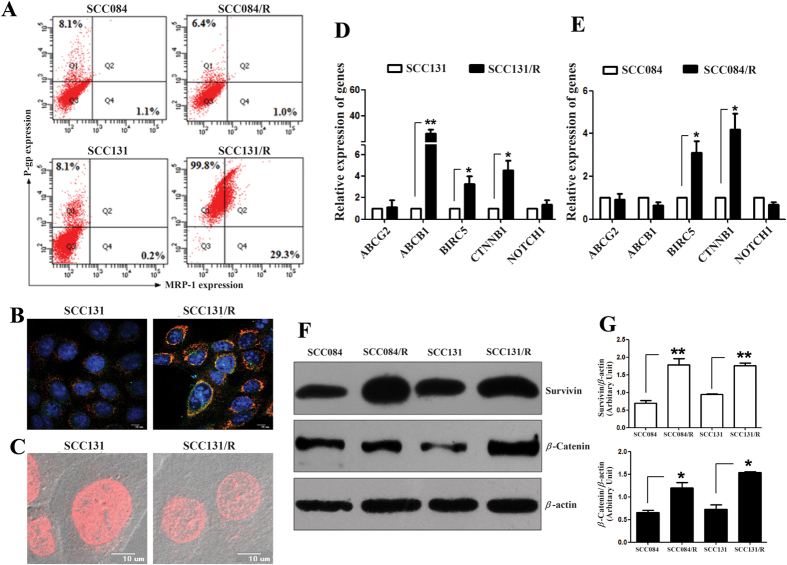
Changes in molecular properties of cisplatin-resistant cells (SCC131/R and SCC084/R) compared to their parental (SCC131 and SCC084) OSCC cells. (**A**) P-glycoprotein and MRP1 expression were significantly increased in SCC131/R cell line compared to the parental SCC131 cells but not in SCC084/R. Cells were stained with P-gp mouse monoclonal UIC2 followed by anti-mouse PE-conjugated secondary antibodies along with FITC-conjugated mouse monoclonal MRP-1 antibody and evaluated using FACS. (**B**) Representative image of confocal microscopy for P-gp and MRP expression in SCC131 and SCC131/R cells. Cells were stained same as FACS –staining except P-gp mouse monoclonal antibody C219 for Confocal microscopic analysis. (**C**) Confocal microscopic images showing the doxorubicin accumulation was significantly reduced in SCC131/R cells compared to SCC131 cells. After seeding a day before cells were incubated with doxorubicin (10 μM) for 1 h at 37 °C then washed with PBS before glycerol mounting and analysed immediately for intracellular accumulation of doxorubicin using confocal microscope. (**D,E**) mRNA expression level of ABCG2, ABCB1 (P-gp), BIRC5 (survivin), CTNNB1 (β-catenin) and NOTCH1 in both the resistant (SCC131/R: D and SCC084/R: E) cells compared to their parental (SCC131: D and SCC084: E) cells. Total RNA isolated was reverse transcribed and the cDNA subjected to qRT-PCR using gene specific forward and reverse primers. Relative expression values were normalized to those of 18 s rRNA. (**F**) Western blot images showing the survivin and β-catenin protein expression were significantly increased in both resistant (SCC131/R and SCC084/R) cells compared to their parental cells (SCC131 and SCC084). Whole cell-lysates from different cell lines were subjected to western blot using specific antibodies. Relative expression values were normalized to those of β-actin. (**G**) Densitometric quantification of survivin and β-catenin protein band in resistant and parental cells. Immunoractive bands were quantified and expressed as the ratio of each band density to after normalization to loading control (β-actin) band density. For (**D,E,G**), data represent three independent experiments and expressed as mean ± SE. For (**A–C,F**), images are representative of three independent experiments. *p < 0.05, **p < 0.01, ***p < 0.001.

A diverse range of molecular mechanisms have been implicated in acquired-drug resistance^8^,^9^. The overexpression of drug efflux pump (ABC-transporter) is frequently associated with drug resistance phenotype^8^. To investigate the molecular mechanism of acquired resistance to cisplatin in these cell lines SCC084/R and SCC131/R, first we checked for differential changes (if any) in expression of different ABC-transporters. We have found that P-gp (ABCB1) and MRP1 (ABCC1) expression were significantly increased in SCC131/R cells compared to the parental SCC131 cells but the similar observation was not true for SCC084/R and SCC084 cells when analysed by using the FACS, confocal microscopic analysis (for both P-gp and MRP1) and quantitative real-time PCR (for P-gp) (Fig. 3A–D). Doxorubicin accumulation study corroborates our results and revealed that doxorubicin accumulation was significantly reduced in SCC131/R cells compared to SCC131 cells (Fig. 3E). However, another important transporter ABCG2 was not changed significantly in both the cisplatin-resistant cell lines as we have checked this at mRNA level (real-time PCR) (Fig. 3B,C).

Next we screen for some pathway specific proteins like survivin (encoded by *BIRC5*), β-catenin (encoded by *CTNNB1*) and NOTCH1 in these cisplatin-resistant cell line. Remarkably, we have found that both survivin and β-catenin were significantly up-regulated at the mRNA and protein level in both the cisplatin resistant cells (Fig. 3D–G) but no significant change was found in NOTCH1 (mRNA level) expression (Fig. 3D,E). All these results suggested that although the survival mechanisms are upregulated by increasing the level of survivin and β-catenin in both the resistant cell lines but the overall resistance mechanisms are not same for two different cisplatin-resistant OSCC cell lines.

### Cisplatin-resistant OSCC cells demonstrate enhanced cancer-stem-cell (CSC)-like property

**Figure 4:**
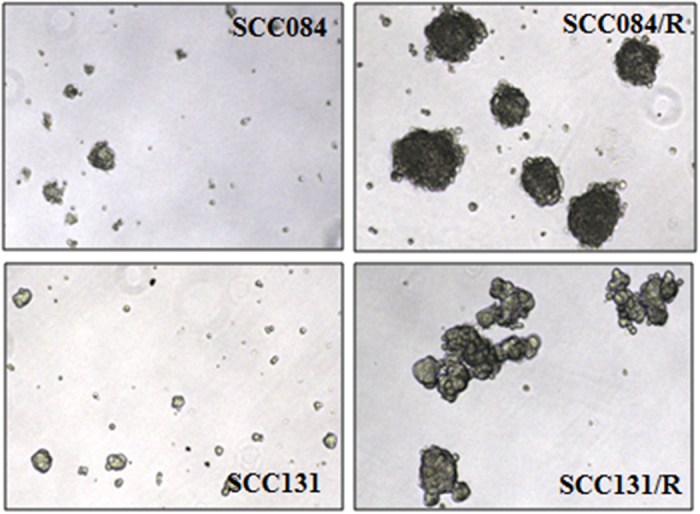
Cisplatin-resistant (SCC131/R and SCC084/R) cells show increased spheroid formation and self-renewal property. Cells were seeded on ultra low attachment plates in sphere forming media. The sphere-forming ability of cells was evaluated after 10 days under microscope (4Χ magnification). Upper panel: SCC084 and SCC084/R cells; Lower panel: SCC131 and SCC131/R cells. These are the representative images of three independent experiments.

Since, stem-cell-like cancer cells are believed to be coupled with drug-resistant property^9^,^15^,^16^; here we tried to find out that if any stem-cell-like feature present in these cisplatin-resistant OSCC cell lines. The results of the sphere forming assay revealed that the tumour sphere forming-ability was significantly increased in cisplatin-resistant SCC084/R and SCC131/R cells compared to parental SCC084 and SCC131 cells. The number and size of the sphere was increased drastically in both cisplatin-resistant cell lines within 7 days (Fig. 4). The consequences imply that cisplatin-resistant cells are enriched in self-renewing cells as evidenced by their greater ability to survive in tumourosphere condition.

**Figure 5:**
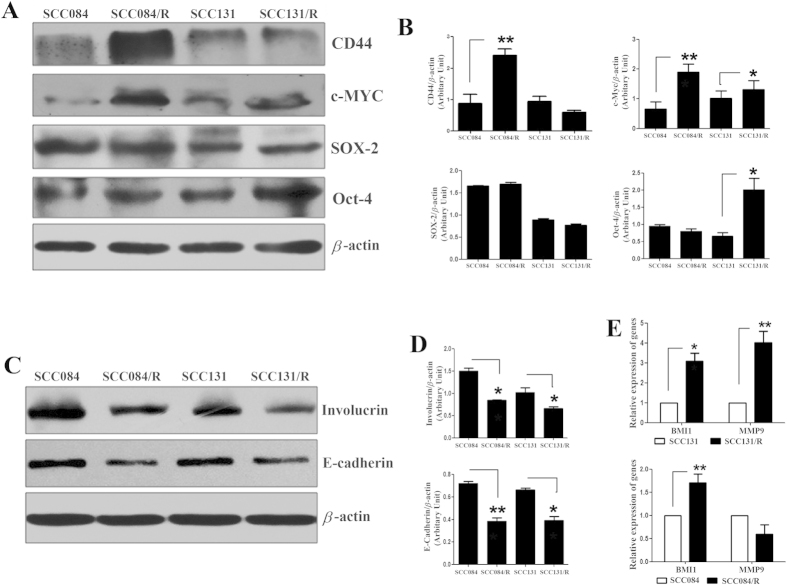
Induction of cancer stem-cell markers and EMT in cisplatin-resistant OSCC cells, SCC131/R and SCC084/R. (**A**) Representative images for changes in cancer stem-cell markers, CD44 c-Myc, Sox2 and Oct-4 expressions in cisplatin-resistant and parental cells. Cell lysates from (SCC131/R and SCC084/R) and parental (SCC131 and SCC084) were subjected to western blot analysis. Indicated protein specific antibodies were used to evaluate the level of cancer stem-cell markers. Relative expression values were normalized to those of β-actin. Western blot images are representative of three independent experiments. (**B**) Densitometry analysis of specific marker protein bands after normalization against the respective loading controls (β-actin) in resistant and parental cells. Data represent the mean ± SE calculated from three independent experiments. (**C**) Western blot images for EMT marker, E-cadherin protein expression was significantly decreased in cisplatin-resistant to parental cells whereas the differentiation marker Involucrin protein was also reduced significantly in cisplatin-resistant cells. Relative expression values were normalized to those of β-actin. Images are representative of three independent experiments. (**D**) Densitometry analysis of protein bands after normalization against the respective loading controls (β-actin) in resistant and parental cells. Data represent the mean ± SE calculated from three independent experiments. (**E**) mRNA expression level of BMI1 and MMP9 in both the resistant (SCC131/R and SCC084/R) cells compared to their parental (SCC131 and SCC084) cells. Total RNA isolated was reverse transcribed and the cDNA subjected to qRT-PCR using gene specific forward and reverse primers. Relative expression values were normalized to those of 18 s rRNA (endogenous control). Data represent three independent experiments and expressed as mean ± SE. *p < 0.05, **p < 0.01, ***p < 0.001.

Moreover, the western blot analysis also revealed resistant cells displayed increased expression of cancer-stem-cell markers than the parental cells. CD44 and c-Myc protein expression were significantly increased in SCC084/R cells while c-Myc and Oct-4 proteins were significantly increased in SCC131/R cells compared to SCC084 and SCC131 respectively (Fig. 5A,B). However, Sox-2 protein expression didn’t show any significant change in both the resistant cell lines (Fig. 5A,B). All these results revealed that both the cisplatin-resistant OSCC cells are significantly associated with induced CSC-like characteristics compared to their parental counterpart.

### Resistant cell lines display induced EMT (epithelial-mesenchymal transition)

**Figure 6:**
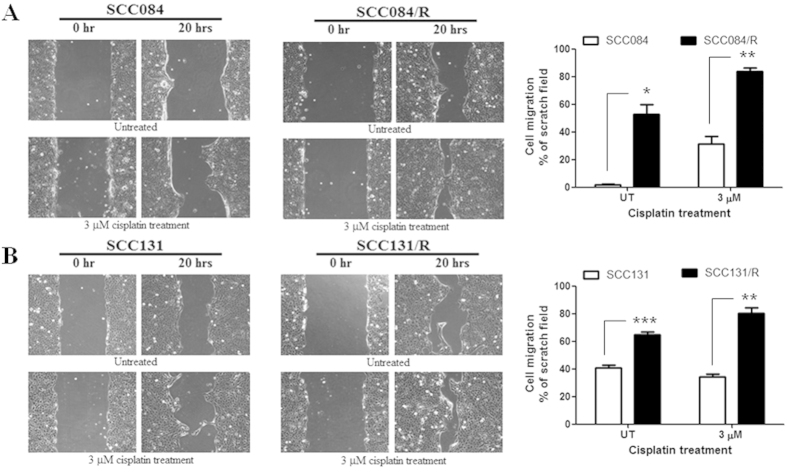
Cellular migration was enhanced remarkably in cisplatin resistant cells. The migration potentiality of resistant and parental OSCC cells was evaluated under microscope (10Χ magnification) by scratch assay in presence or absence of cisplatin. Drug treatment was done for 24 hrs before the scratches made. (**A**) Upper left panel shows representative images of scratch assay using SCC084 and SCC084/R cells respectively taken at 0 h and 20 h after the scratch made in presence or absence of cisplatin treatment. The upper right panel shows the quantification of cell migration (% of scratched areas) after 20 hrs calculated using Image J in SCC084 and SCC084/R cells. (**B**) Lower left shows representative images of scratch assay using SCC131 and SCC131/R cells respectively taken at 0 h and 20 h after the scratch made in presence or absence of cisplatin treatment. The lower right panel shows the quantification of cell migration (% of scratched areas) after 20 hrs calculated using Image J in SCC131 and SCC131/R cells. Images are representative of three independent experiments. In the bar-diagram the data expressed as mean ± SE. *p < 0.05, **p < 0.01, ***p < 0.001.

Augmented sphere-forming ability and CSC-marker expression in the cisplatin-resistant cells as well as distinct change in cellular-morphology lead us to speculate whether there is any change in the expression of EMT-markers^15^,^16^,^17^ and hence in cellular migration. To confirm the induction of EMT in cisplatin-resistant cells, we analyze the expression of E-cadherin^15^,^17^ protein using western blot in comparison to parental-sensitive cells. The level of E-cadherin expression in resistant cells was significantly decreased compared to that of parental cells (Fig. 5C,D). Another protein involucrin whose expression is known to correlate with the degree of differentiation^18^, is also considerably reduced in cisplatin-resistant cell lines compared to parental cell lines (Fig. 5C,D). Another important EMT-related gene, BMI1^15^,^16^ and MMP9^15^,^16^,^19^ expressions were also evaluated at mRNA level by qRT-PCR in these cell lines. The results revealed that BMI1 expression was significantly increased in both the resistant cell line whereas MMP9 expression was remarkably increased only in SCC131/R cells compared to respective parental cells (Fig. 5E). All these results suggested that in both the cisplatin-resistant cells EMT-phenotype are significantly increased compared to their parental counterpart. To substantiate our result further we intended to check the potential ability of cell migration in these cell lines by *in vitro* scratch assay. Interestingly, we discovered that irrespective of drug treatment the cellular migration was significantly increased in cisplatin-resistant cell line compared to their parental-sensitive cells (Fig. 6). Therefore, the overall culmination revealed that cisplatin-resistant cell lines demonstrate significant induction in EMT as well as cellular-migration ability.

### Cisplatin-resistant oral cancer cell lines exhibit distinct miRNA expression pattern with a complex target gene regulatory-network

**Figure 7:**
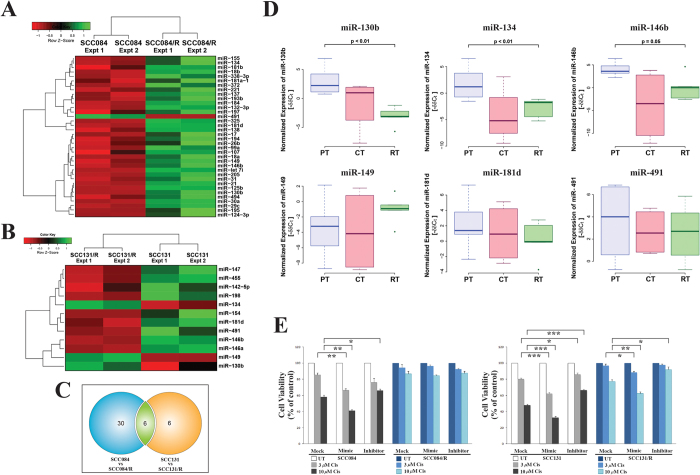
Cisplatin-resistant specific miRNA selection from cisplatin-resistant and parental OSCC cell lines and validation by means of *in vitro* (altering the endogenous miR-level) and *in vivo* (using clinical samples) analysis. (**A**) Cluster of 36 miRNAs (listed on the right) which show significant differences (*p* ≤ 0.05) in the expression profile in SCC084/R cells compared with that in SCC084 cells. (**B**) Cluster of 12 miRNAs (listed on the right) which show differential expression profile in SCC131/R cells compared with that in SCC131 cells. In figure (**A**,**B**) heatmap was generated using the normalized expression values (z-score of ∆C_T_) of those significantly (*p* ≤ 0.05) differentially expressed miRNAs. Each column represents the expression values of miRNAs from an independent experiment (two different passages of the cell line were used and displayed as Expt 1 and Expt 2) with three technical repeats. (**C**) Venn diagram on total number (in parenthesis) and overlapping number of differentially expressed genes calculated in the cell line pairs consisting the cisplatin resistant to their parental cells. (**D**) Box plots of endogenous levels of six resistance specific miRNAs in primary tumour (PT, n = 6), neoadjuvant chemotherapy treated tumours (CT, n = 4) and recurrent rumours (RT, n = 5) in patients with OSCC. Total RNA was isolated and subsequently used for quantitative real time-PCR. The negative values of miRNA expression (−∆∆C_T_) were plotted after normalisation to the expression of U6 (endogenous control) and to the expression of miRNA in adjacent normal tissues. (**E**) MTT assay was used to validate the role of miR-134 in resistance development, the resistant (SCC084/R; SCC131/R) and parental (SCC084; SCC131) cell lines through altering its endogenous level of miR-134 expression. Cells were transfected either with miRNA mimic or miRNA inhibitor or control (Mock) using Lipofectamine 2000. Cells were subsequently treated with cisplatin after transfection and incubated for 48 hrs. Then cell viability was measured to assess the cisplatin mediated cytotoxicity in miR-134-overexpression and -inhibition condition in these OSCC cell lines. Data represent three independent experiments and expressed as mean ± SE. *p < 0.05, **p < 0.01, ***p < 0.001.

Next our specific aim was to identify the critical miRNAs those are involved in the development of cisplatin-resistant phenotype in oral cancer. We compared 52 miRNAs which are found to be deregulated in head and neck cancer malignancies (Supplementary Table S3). We extracted the list of miRNAs those are revealed by 6 different studies with tumour samples of head and neck cancer patients from PhenomiR Database v2.0 (as on May 2014)^20^. From the database we have found that, 15 miRNAs are downregulated whereas 37 miRNAs are upregulated among those 52 deregulated miRNAs in HNSCC (Supplementary Table S3). Two parallel sets of cisplatin-resistant and parental cell lines, (SCC084/R and SCC084) and (SCC131/R and SCC131) were profiled to identify miRNAs which are associated with the development of drug resistance in OSCC. The miRNA profiling of SCC084/R compared to SCC084 cells revealed that 36 miRNAs were differentially expressed (*p* ≤ 0.05) and shown in the Fig. 7A and Supplementary Table S4. Conversely, the profiling also disclosed that 12 miRNAs are differentially expressed (*p* ≤ 0.05) in SCC131/R cells compared to SCC131 cells as summarised in the Fig. 7B and Supplementary Table S4. Finally, we have established 6 miRNA signatures (miR-130b, miR-134, miR-149, miR-491, miR-181d, miR-146b) which are significantly (*p* ≤ 0.05) differentially expressed and common in both the cisplatin resistant cell lines (SCC084/R and SCC131/R) compared to their parental cell lines (SCC084 and SCC131) (Fig. 7C and Supplementary Table S5). Next, to confirm our *in vitro* cell line data, the expression status of these signature miRNAs were checked in few patient sub-groups comprised of primary tumours (PT; n = 6), neoadjuvant chemotherapy treated tumours (CT; n = 4) and recurrent tumours (RT; n = 5) in patients with OSCC (see Materials and Method section). Detailed patient history is provided in the Supplementary Table S6. Significant changes (significant p values given in Fig. 7D, Supplementary Table S7) in miRNA expression were observed in recurrent tumours when compared to primary tumours in patients with OSCC. Moreover, we confirmed the role of miR-134 in resistance development (randomly selected miRNA from those six signature miRNAs) through altering its endogenous expression by using its mimic and inhibitor in OSCC cell lines. The results revealed that both the parental cell lines (SCC084 and SCC131) showed significant increase in cisplatin-sensitivity and cisplatin-resistance along with overexpression and inhibition of endogenous miR-134 expression respectively in these OSCC cell lines (Fig. 7E). Similarly SCC131/R cell line displayed the significant changes in cisplatin-sensitivity/resistance whereas SCC084/R did not display any notable difference when we altered the level of miR-134 expression by using its mimic and inhibitor compared to the mock.

**Figure 8:**
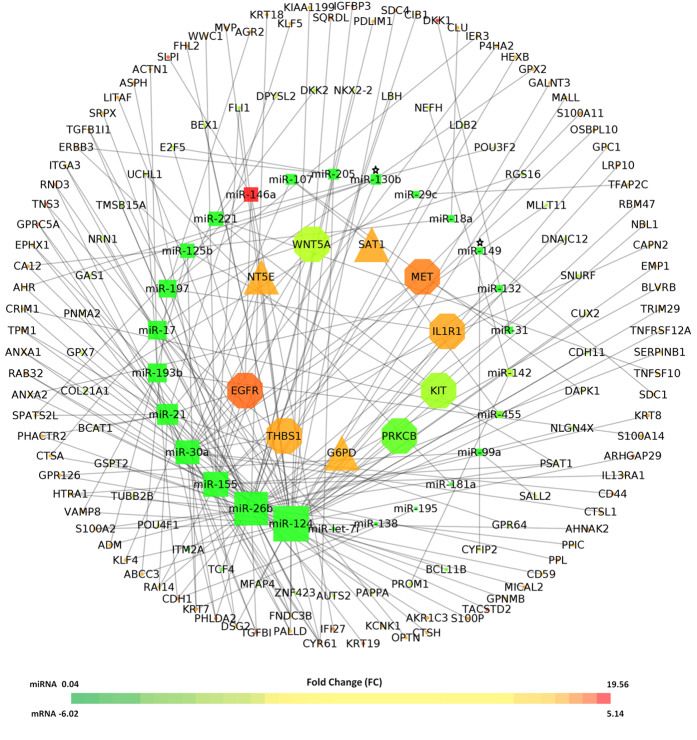
Regulatory network of deregulated miRNA-mRNAs in cisplatin resistant condition. Size of the nodes in the network are weighted based on degree (number of mRNA targets) of 26 deregulated miRNA (square shape) and involvement of 144 mRNA target genes/proteins in signalling/metabolic pathways where signalling cross-talk proteins are shown as octagon shape and rate limiting enzymes in triangle. Colour of the nodes is dependent on their expression fold change (fold change: >2.0 x; *p* ≤ 0.05). Commonly deregulated miRNA’s whose target genes exhibit significantly higher deregulation (fold change: 2.0 x; *p* ≤ 0.05) expression in multi cancer cell lines are marked by *.

Further, we have explored the expression status of the target mRNA/genes (see material and method section) using the miRTarBase and ONCOMINE database^21^,^22^, of the deregulated miRNAs (36 unique and 6 common) observed in SCC084/R and SCC131/R cell lines. However, only 26 out of the 40 miRNAs (miR-181d and miR-146b were excluded as they displayed opposite expression status in SCC084/R and SCC131/R) yielded experimentally known target mRNAs. 100 experimentally known target genes are found to be upregulated while 44 are downregulated in multiple types of cancer cell lines (Supplementary Figure S1). Most of these target genes are involved in catalytic, binding and transcriptional functions (Supplementary Figure S2). A large fraction (20%) of the target genes is found to be transcription factor indicating probable silencing of critical factors under cisplatin resistance scenario. Figure 8 shows miRNA-mRNA regulatory network, involving deregulated (up/down) miRNAs and their deregulated targets (fold change: >2.0x; *p* ≤ 0.05) in cisplatin resistant scenario. 7 signalling cross-talk proteins (octagon shape) and 3 rate limiting enzymes (triangle shape) are found to be deregulated under cisplatin resistance in various multi cancer cell lines. Interestingly, ATP-binding cassette (ABC) transporter family, Baculovirus Inhibitor of apoptosis Repeats Containing genes (BIRC family), are also found to be upregulated and WNT/β-catenin pathway related genes (DKK1, DKK3, GSK3β, TCF4 etc) are also found to be altered significantly in multiple cancer cell lines (Fig. 8 and Supplementary Table S8) under cisplatin resistant condition. Similarly, CSC marker CD44 expression is also found to be upregulated. Expression fold changes of the target genes of 6 commonly deregulated miRNAs are provided in Supplementary Table S9. All these results revealed the resistance specific signature of miRNAs which might have an intricate role in the development of cisplatin-resistant oral cancer cells with CSC and EMT-type properties.

## Discussion

One of the greatest impediments in improving the survival rates of head and neck cancer patients after single/multimodality (surgery, chemotherapy and radiotherapy) treatment has been a lack of understanding of the mechanisms by which residual cells (resistant) survive from the heterogeneous cell population of the tumour after treatment. On the other way, the presence of cancer stem cells in association with drug resistance and EMT in these heterogeneous (cellularly and molecularly) population are frequently overruled by the treatment regime and thus responsible for disease relapse and metastasis^15^,^16^. Therefore, the understanding of the resistance mechanisms to chemo-radiation-therapy is indispensable and a systemic attempt is needed to improve the outcome of the head and neck cancer patients. Recent reports suggested that the short/long term exposure of cisplatin treatment leads to the enrichment of residual cells with cancer stem-like cells in association with EMT in different solid tumours^23^,^24^. The novelty of the present study is the use of cisplatin-resistant cell lines of two different parent cell lines originated from recurrent and primary tumours of OSCC patient, to dissect out the cellular mechanisms responsible for survival and the subsequent behaviour of cisplatin-resistance in OSCC and here we establish the association of CSC specific markers, EMT and cellular migration with the survival mechanism of resistant OSCC cells. Furthermore, in the present study, we compared the differential profiles of miRNAs between cisplatin-resistant and their parental OSCC cell lines and try to correlate with their functionality.

In this study, we have developed two cisplatin-resistant (SCC084/R and SCC131/R) cell lines from their parental (SCC084 and SCC131) cell lines and characterised them in terms of their proliferative, clonogenic survival ability, apoptotic potential, and cell cycle distribution. Apart from the fold change in cisplatin-resistance (R index; Supplementary Table S2) compared to respective parental cell line we have found that as SCC084 which was originated from the recurrent tumour has 3.5 fold higher IC50 value than SCC131 originated from primary tumour and in turn, all these reflect the actual pathological scenario. As cancer cells undergo adaptive changes, molecular characterisation of these cells was aimed to identify their survival mechanisms after resistance development in these OSCC cells. We have found that SCC131/R resistant cells were exacerbating drug resistance through overexpression of ABC-transporter efflux proteins, P-gp (ABCB1) and MRP1 (ABCC1) whereas SCC084/R might have the other resistance mechanism. Therefore, next we screened for some survival signalling pathway specific proteins like survivin (encoded by *BIRC5*), β-catenin (encoded by *CTNNB1*) and NOTCH1, those were found to be up-regulated in different malignancies and sometimes showed correlation with poor prognosis^6^,^9^,^16^. In our present investigation we observed a significant up-regulation of survivin and β-catenin in the cisplatin-resistant SCC084/R and SCC131/R cells compared to parental SCC084 and SCC131 cells. Whereas NOTCH1 gene expression showed no change in these two resistant OSCC cell lines suggesting the possibilities of other molecules in Notch signalling to be involved in this process. Accumulating evidences also revealed a strong correlation with the increased expression of these proteins, survivin and β-catenin with the poor progression of patients with solid tumour malignancies^25^,^26^,^27^,^28^. The survivin is highly tumour-specific molecule and its up-regulation in tumours caused the poor response to chemo-radiation therapy^25^. β-catenin is one of the key modulator of cancer cells and responsible for creating a suitable niche for cancer progression by modulating tumour-microenvironment^28^. β-catenin directly regulates the expression of survivin and associated with the characteristic signature of stem-like cell populations in cancer^26^,^27^,^28^. Computational analysis of the deregulated miRNA-mRNA network suggests deregulation of multiple important target genes in sync with their regulatory miRNA expression status. In support of the experimental observations of this study, upregulation of ATP-binding cassette (ABC) transporter family, Inhibitors of apoptosis containing genes (BIRC family), and CSC marker CD44 expressions are also suggested via computational analysis.

In the present study, we have found a significant enrichment of CSC-like cells with an increase in their self-renewal and tumourosphere formation capacity in cisplatin-resistant SCC084/R and SCC131/R cells. Equally, an increase in the CSC markers (CD44, c-Myc, and Oct-4) in these resistant cells demonstrated an enrichment of cells with CSC-like nature further substantiating our finding at functional protein level. In several reports CD44, c-Myc and Oct-4 are predicted as a poor prognostic marker in different malignancies^24^,^29^,^30^,^31^. In our observation we found that CD44 expression was considerably high in recurrant-origin SCC084/R cell line compared to primary tumour-origin SCC131/R cell line. Similar observation also substantiates our data and revealed that CD44 expression is correlated with local recurrence in oral cancer^24^,^30^, whereas c-Myc overexpression predicts aggressive transformation and poor outcome in lymphomas and other malignancies^30^,^31^. Another study on breast cancer reported that Oct-4 promote the EMT of CSCs and are associated with poor prognosis^32^. In addition, significant reduction in expression of E-cadherin and involucrin in association with significant increase BMI1 and MMP9 expression in cisplatin-resistant SCC084/R and SCC131/R cells further authenticate our observation on spindle-shaped mesenchymal-like cellular transformation towards EMT as well as enrichment of CSC-like behaviour. All these molecular expression has strong correlation with EMT, invasion and metastasis and overall poor survivality in different solid tumour malignancies^15^,^17^,^18^,^19^. Increased level of BMI1 has been considered as a poor prognostic marker in oral cancer^16^,^24^. In concordance with our study, a very recent study has also reported that the triple (Taxol, platinum, 5-fluorouracil) drug-resistance, mediated through P-gp (MDR-1), MRP-2 and survivin, is coupled with the acquisition of cancer stem cell behaviour in association with induced EMT in head and neck cancer and they suggested that acquired drug-resistance is probably achieved by induction of these molecules through multiple EMT and CSC-mediated pathways and showed significant correlation with the prognostic outcome of the patient^24^. Therefore, the results from the present investigation (experimental studies and bioinformatic studies) concludes that Wnt-pathway/β-catenin-survivin axis might play a significant role in development of resistance phenotype in both the cell line by up-regulating ABC-transporter P-gp, MRP1 together with enrichment of the CSC-like cells (CSC markers CD44, c-Myc, Oct-4 overexpression) in conjunction with inducing EMT (through E-cadherin down-regulation and BMI1, MMP9 up-regulation) and cellular migration.

A number of studies have been recently carried out to elucidate the pattern of miRNA expression contribute to phenotypic outcome in OSCC patients^11^,^12^,^33^. In our present study, we demonstrated differential expression profile of miRNA in cisplatin-resistant oral squamous cell carcinoma cell lines. In the present study we profile only those miRNAs (52 miRNAs) which are found to be dysregulated in HNSCC patient (PhenomiR database v2.0)^20^ presuming that these miRNAs have significant role in tumourogenesis process in oral cancers. The results revealed that SCC084/R cell line having recurrent-tumour origin show dysregulation in 36 miRNAs compared to parental SCC084 cell line (Supplementary Table S4). Among them 35 miRNAs are significantly (p ≤ 0.05) down-regulated and only one miRNA (miR-491) is up-regulated in SCC084/R cells. Whereas 12 miRNAs (3 miRNAs down-regulated and 9 miRNAs up-regulated) are differentially expressed (p ≤ 0.05) in SCC131/R cell line having primary-tumour origin when compared to SCC131 cell line (Supplementary Table S4). There are 6 miRNAs, miR-130b, miR-134, miR-149, miR-491, miR-181d, miR-146b, which are common in both the resistant cells. However, the miRNA expression pattern is not exactly same in both cases (Supplementary Table S5). Although, the miR-181d and miR-146b are significantly changed in both the resistant cell line, both the miRNAs are up-regulated in SCC131/R cells while these are down-regulated in SCC084/R (Supplementary Table S5). These differences in expression pattern may be due to the distinct resistance mechanism in these two cell lines originated from distinct tumour-type (primary and recurrent) of OSCC patients (Supplementary Table S1). Moreover, recent studies also revealed that the different cancer etiology may affect miRNA expression machinery differently^28^. In our study, the recurrent (more aggressive) patient sub-group showed significant changes in resistance specific miRNA expressions when compared to those of primary tumours in OSCC patients. However, the point to be noted that the differential factors like etiological factors, primary site, stage, grade, and treatment regimen were different among patient sub-groups.

Previous studies found that miR-130b is significantly deregulated in various tumour types including gastric cancer, pancreatic cancer, and endometrial cancer^34^,^35^,^36^. Moreover, a recent study revealed that in pancreatic cancer miR-130b could be a biomarker to predict the prognosis and progression of the patients as the down-regulated miR-130b was correlated with worse prognosis, lymphatic invasion and distant metastasis in pancreatic cancer^33^. Consistent to our results, earlier studies on miR-134 also revealed the similar results, comparatively low expression of miR-134 correlates with the invasive potential and EMT phenotype of HNSCC^37^ and non small cell lung carcinoma (NSCLC) cells^38^. Functional assays showed that miR-134 inhibits EMT in NSCLC cells and Forkhead box protein M1 (FOXM1), a potential metastasis promoter, was a direct and functional target of miR-134^38^. Also low expression of cell-free miR-134 in lung adenocarcinoma-associated metastatic malignant pleural effusion (MPE) is correlated with poor survival^39^. Recent genome-wide screening identified that miR-134 acts as a metastasis suppressor by targeting integrin-β1 in hepatocellular carcinoma^40^. Similarly studies also revealed that miR-149 is correlated with cell migration, invasion and EMT by targeting FOXM1 in colorectal cancer^41^, NSCLC^42^. In addition, down-regulated miR-149 has been shown to be associated in the crosstalk between tumour cells and stroma modulating the tumour-microenvironment and responsible for the tumour initiation and growth in a very recent study^43^. Cancer-associated fibroblasts (CAFs) in tumour stroma enhanced EMT and the CSC-like properties of gastric cancer cells in a miR-149-IL-6-dependent manner^43^. On the other hand, IL-6 has a very important role in development of drug resistance in cancer and correlated with the poor disease-outcome^9^. Corroborating our findings this has been reported that the expression of miR-146b (low or high) in cancers has been associated with prognosis of the disease. Up-regulation of miR-146b-5p in human tumours was found to be associated with aggressiveness and poor overall survival in NSCLC^44^, human papillary thyroid carcinoma^45^, and in muscle-invasive bladder cancer (MIBC)^46^. In contrary it has also been found that low expression of miR-146b-5p predicts poor prognosis and poor outcome of large B-cell lymphoma treated with cyclophosphamide, doxorubicin, vincristine and prednisone^47^. Similarly, there is an evidence of low level of miR-146b-5p responsible for the prometastatic process through AUF1 gene expression and miR-146b-5p expression positively regulate the epithelial markers (E-cadherin, EpCAM) and repress the mesenchymal markers (N-cadherin, vimentin, twist2 and ZEB1^48^. Consequently, the level of miR-181d expression also revealed as a putative biomarker for lymph-node metastasis of oral cancer^49^, and correlated with clinicopathological features in ovarian cancer^50^ and suggested as a predictive biomarker for glioblastoma^51^. miR-181d was found to be up regulated in breast cancer compared to normal adjacent tumour tissues^52^. However, miR-491-5p displayed a significantly low level of expression in pancreatic cancer cell and mediated cell apoptosis by targeting both Bcl-xL and TP53^53^. In consistent with our results, a report also suggested that the level of miR-491-5p expression correlated inversely with GIT1, which in turn is correlated with lymph node metastasis and tumour grade in OSCC clinical samples and finally they suggested miR-491-5p and GIT1 as biomarkers for prognosis in this cancer^54^. Keeping in mind the background evidences and in light of the current results, (as in Supplementary Table S9) we can suggest that the microenvironment required for the development of these OSCC resistant cell lines with all these particular phenotype is probably created by these resistance specific miRNA expression signature patterns through modulating their target gene expression in OSCC.

In conclusion, in the present study we have demonstrated 6 differentially expressed miRNAs signature pattern in two cisplatin-resistant OSCC cell lines those were enriched with the cancer-stem-cell-like characteristics sometimes in association with drug efflux transporters mediated drug-resistance through inducing EMT and cell migration. In addition, we can envisage that these cellular transformation and miRNAs expression pattern might have some correlation with the patient outcome. Consequently, these expression patterns most likely anticipated as useful prognostic biomarker for oral cancers.

## Materials and Methods

### Cell lines and induction of cisplatin-resistance in OSCC cells

The human oral squamous cell carcinoma cell lines, SCC084 and SCC131 were purchased from Dr. Susanne Gollin (University of Pittsburgh, USA) (Supplementary Table S1)^14^. All OSCC cells were grown in DMEM/F12 (1:1) (Gibco, Life Technologies, Carlsbad, CA, USA) supplemented with 10% FBS (Gibco, Life Technologies), 1% L-glutamine (Gibco, Life Technologies), 1% antibiotic mixture (Penicillin-Streptomycin-Neomycin or PSN; Gibco, Life Technologies) and 0.006% Gentamicin (Gibco, Life Technologies) at 37 °C in a humidified 5% CO_2_ incubator.

Cisplatin [*cis*-diamminedichloroplatinum (II), CDDP] was obtained from Sigma-Aldrich and dissolved in PBS. Aliquots were stored at 4 °C and used for 7 days. In accordance with previously described methods^55^ cisplatin resistant SCC084/R and SCC131/R (pooled clones) were developed from their parental cell line SCC084 and SCC131 respectively by gradual incremental doses of cisplatin administration in cell culture medium in subsequent passages for about 6–8 months. Thereafter SCC084/R and SCC131/R cells were maintained in presence of 1 μg/ml (3 μM) cisplatin in cultured medium for 48 h in every alternate passage. In case of resistant cell lines, any experiment was set up with cells when those were grown in drug-free culture medium. All the resistant were cultured in the same conditions as parental OSCC cell lines.

### Cytotoxicity assay

Cytotoxic effect of cisplatin was measured by MTT assay. Cells were seeded in 96-well plates at a density 2 × 10^4^ for SCC084 and SCC084/R and 1 × 10^4^ for SCC131 and SCC131/R cells per well. The cells were incubated overnight at 37 °C to allow adhere and recovery. Increasing concentrations of cisplatin was administered for each cell line and incubated for different time points like, 24 h, 48 h and 72 h with 5% CO_2_ at 37 °C. After completion of incubation, 4 μl of a 5 mg/ml solution in PBS of MTT dye (3-[4,5-dimethylthiazol- 2-yl]-2, 5-diphenyltetrazolium bromide; Sigma) was added to each well and incubated for 4 h at 37 °C. The media discarded and the resulting violet formazan precipitate was dissolved in 100 μl of DMSO. The colour absorbance was recorded at 540 nm by microplate reader (Multiskan Ex). The control value corresponding to untreated cells was taken as 100% and the viability of treated samples were expressed as a percentage of the control. The IC_50_ values were determined as the concentration that reduced cell viability by 50%. The degree of resistance is evaluated in terms of resistance index, R (R = IC_50_ values of resistant cells/ IC_50_ values of parental cells).

### Clonogenic survival assay

The sensitivity of SCC084, SCC131, SCC084/R and SCC131/R cells to cisplatin was measured using the clonogenic assay or colony formation assay based on the ability of a single cell to grow into a colony. For each cell lines 500 cells were seeded in each well of a 6 cm dish and allowed to adhere overnight at 37 °C and treated with different doses of cisplatin for 24 h. Then medium was changed to drug free medium and cells were allowed to grow into colonies within next 7–14 days. Colonies were analysed microscopically and fixed and stained with 0.2% methylene blue (Sisco Research Laboratories, Mumbai, India) in PBS and washed with water. The colonies were then counted using Image J software and the number of colonies was calculated as mean of three independent experiments.

### Cell cycle analysis

2 × 10^5^ cells were seeded in 60 mm dish and treated with different doses of cisplatin for 72 h. Cells were collected by tripsinization and washed twice in PBS, fixed in 70% chilled ethanol by adding dropwise into the samples while vortexing and stored at 4 °C until analyzed. Immediately before analysis, cells were washed twice in PBS, and incubated with 250 μg/ml RNase A (Invitrogen) for 30 min at room temperature, followed by staining with PI (Sigma) at 20 μg/ml final concentration for 30 min incubation in dark. The cell cycle distribution and percentage of apoptotic cells were analysed using a FACS analyser (BD LSRFortessa). Ten thousand cells were analysed for each sample. Appropriate gating was used to select the single-cell population. The percentage of cells in sub-2n phase was determined using software (BD FACSDiva v6.0).

### Annexin V-PI binding assay

Apoptosis was determined by annexin V/ PI staining with the apoptosis detection kit (BD Pharmingen). 1 × 10^5^ cells were treated with different doses of cisplatin for 72 h. Cells were collected by tripsinization and washed twice in PBS. Cells were stained with Annexin V- FITC and PI at room temperature for 15 min in the dark and analyzed by FACS using two-colour FACS analysis (BD LSRFortessa).

### Flow Cytometric analysis of putative ABC-transporters

Cisplatin-resistant and parent cells (1 × 10^6^) were collected by trypsinization and washed twice in PBS containing 1% FBS and pellet by centrifugation at 1500 rpm for 3 min. Dual staining for P-glycoprotein (P-gp) and Multi-drug Resistance-associated Protein-1 (MRP-1) was carried out. Cells were incubated with mouse monoclonal P-gp antibody (UIC2) (Santa Cruz Biotechnology) and then with PE-conjugated secondary antibody and subsequently by FITC-conjugated mouse monoclonal MRP-1 antibody (BD Biosciences). Cells were washed briefly and resuspended in PBS for subsequent analysis. Samples were acquired (10,000 cells) and analysed by FACS (BD LSRFortessa) and the percentage of P-gp and MRP-1 positive cells were calculated.

### Confocal microscopic analysis for ABC-transporters

The levels of P-gp and MRP1 expression were also analyzed by mouse monoclonal anti P-gp antibody (C219) (Calbiochem, USA) and FITC-conjugated mouse monoclonal MRP-1 antibody (BD Biosciences) respectively. Briefly, SCC131/R and SCC131 cells (0.5 × 10^6^ cells) were platted on cover slips for confocal microscopy in labeled groups and cultured overnight followed by fixing of the cells on cover slips with 4% paraformaldehyde. The cells were incubated with P-gp antibody (C219) for two hours. Following incubation, the cells were washed twice with PBS containing 0.1% FBS and re-incubated with PE-conjugated 2° antibody (anti mouse) for another 1 hr. Cells were then subsequently incubated with FITC-conjugated MRP-1 antibody (BD Biosciences) for another two hours. Cells were washed twice with PBS (containing 0.1% FBS) and mounted with antifade mounting media with Dapi-Dabco (Life Technologies) and analyzed in confocal microscope (Andor Spinning Disc Confocal) using Andor iQ 2.7 software for assessing the level of P-gp and MRP1 expression.

### Drug accumulation assay

The fluorescent property of doxorubicin, a good substrate for P-gp, was used for drug accumulation assay^56^. SCC131/R and SCC131 cells (0.5 × 10^6^ cells) were platted on cover slips in 2 ml of complete growth media and left overnight. Fresh media was changed to each group prior to initiate experiment and incubated with doxorubicin (10 μM) at 37 °C for 1 hr. The cells were fixed with 4% paraformaldehyde and washed with PBS containing 0.1% FBS and mounted with glycerol and analyzed immediately for intracellular accumulation of doxorubicin using confocal microscope (Andor Spinning Disc Confocal) and Andor iQ 2.7 software.

### Sphere forming assay

Cells (2 × 10^4^/well) were seeded on ultra-low attachment 6-well plates in serum free DMEM/F12 media supplemented with growth factors. The sphere forming medium was supplemented with 1% B27 supplement (Invitrogen Gibco), 20 ng/ml of human recombinant epidermal growth factor (EGF), 20 ng/ml human basic fibroblast growth factor (b-FGF) and 1% penicillin-streptomycin. The sphere forming ability of the cells was recorded on next 7–14 days. Size and number of spheres were assessed under Leica microscope.

### Cell migration assay

The migratory potential of cells was assessed by the wound-healing scratch assay. 2 × 10^5^ cells were plated in each well in 6-well plates. Cells were treated with cisplatin for 48 h. After completion of incubation a scratch has been made with sterile 200 μl tip and washed cells twice with PBS and restore with drug-free fresh media. Photos were taken of the scratches at 0 h and 20 h using Leica microscope. The percent of cellular migration after scratches made was quantified using Image J software.

### Western blot

Cells plated at high seeding in 10 cm dish were allowed to grow to 90% confluent within 3–4 days. Whole cell lysates having equal protein concentrations were resolved by SDS/PAGE (8–12%) and transferred onto a PVDF (Millipore, Billerica,USA) membrane. Various antibodies used were rabbit monoclonal survivin (Cell Signalling Technology), rabbit polyclonal β-catenin (H-102) (Santa Cruz Biotechnology), rabbit polyclonal E-cadherin (H-108) (Santa Cruz Biotechnology), rabbit polyclonal involucrin (Santa Cruz Biotechnology), mouse monoclonal CD44 (H-CAM, DF1485) (Santa Cruz Biotechnology), rabbit polyclonal c-Myc (Cell Signalling Technology), rabbit polyclonal Sox-2 (Abcam), rabbit polyclonal Oct-4 (Abcam), mouse monoclonal β-actin (Sigma), rabbit polyclonal PARP 1 (Cell Signalling Technology), mouse monoclonal Caspase 3 (E-8, Santa Cruz Biotechnology), mouse monoclonal α-tubulin (Santa Cruz Biotechnology). Bands were detected using super signal west pico chemiluminescent substrate (Thermo Scientific, USA) after treating with HRP-cojugated secondary antibody (Sigma).

### Quantitative Real-Time PCR (qRT-PCR) and RT-PCR

SYBR green qRT-PCR assay was used for miRNA profiling and gene expression analysis by using 7500 Fast real-time PCR system (Applied Biosystem). Total RNA was isolated from cultured cells using the TRIzol reagent (Invitrogen). To analyse the mature miRNA expression, 1 μg total RNA was used for cDNA synthesis by using the 5x miScript HiSpec buffer in miScript II RT kit (Qiagen) according to manufacturer’s instructions. Then PCR product was diluted and 1 ng cDNA was used per reaction for quantitative real-time PCR by using specific miScript primer assays/miRNA specific forward primer (Supplementary Table S10) and miScript Universal primer provided in the miScript SYBR Green PCR kit (Qiagen) following the manufactures protocol. RNU6B (U6) served as an endogenous control for miRNA expression analysis. All reactions were performed in triplicate with two biological repeats. The cycle threshold (C_T_) (considered only the value which is < 35) is defined as the number of cycles required for the fluorescent signal to cross the threshold in qPCR. ∆C_T_ was calculated by subtracting the C_T_ values of U6 from the C_T_ values of miRNA of interest. ∆∆C_T_ was then calculated by subtracting ∆C_T_ of the parental cells (control) from ∆C_T_ of cisplatin-resistant cells (sample). Fold change of each miRNA expression was calculated by comparative method using the equation 2^−∆∆CT^. All the real-time PCR data of miRNA expression were analysed using the Bioconductor Limma packages for two different cell lines separately. Unsupervised hierarchical clustering based upon Euclidean distances was performed on cisplatin-resistant cell lines. Finally, normalized expression values of those significantly (*p* ≤ 0.05) differentially expressed miRNAs were used to draw heatmap using R software (version 3.2.1).

To check the gene expression, cDNAs were synthesized from 1 μg of total RNA using Verso cDNA synthesis kit (Thermo Scientific) according to manufacturer’s protocol. Quantitative RT-PCR was performed by mixing 5 ng of cDNA with SYBR Green PCR Master Mix (Roche) and various sets of gene specific primers (forward and reverse) (Supplementary Table S11). In this case, 18 S rRNA was used as an endogenous reference control. Then these were subjected to RT-PCR quantification by using the 7500 Fast real-time PCR system (Applied Biosystem). All reactions were performed in triplicate. The relative amounts of mRNA were calculated same as miRNA by using the comparative C_T_ method. The results are presented as relative expression of fold change of each mRNA in cisplatin-resistant cells to their parental cells.

### miRNA mimic and miRNA inhibitor transfection

Cisplatin-resistant and sensitive cells (4 × 10^4^ cells/well) were seeded in 24 well plates. Next day OSCC cells were transfected either with 5 nM of miRNA mimic (Qiagen) or 50 nM of miRNA inhibitor (Qiagen) or mock (control) using Lipofectamine 2000 (Invitrogen, Carlsbad, CA) in Opti-MEM reduced serum medium (Invitrogen Life TechnologiesInc., Carlsbad, CA) according to the manufacture’s protocol. After 6 hrs, media replaced with DMEM supplemented with antibiotics and 10% FBS and drug treatment done and incubated for 48 hrs.

### Patient samples

Oral cancer biopsies and surgical tumour samples with adjacent normal tissues were obtained from the hospital section of Chittaranjan National Cancer Institute (CNCI), Kolkata, India. Prior to sample collection, written informed consent was taken from each patient. The study was approved by the Institutional Ethics Committee of CNCI and carried out in accordance with the approved guidelines. For the present study we have selected specific patient sub-groups like OSCC-patients with newly diagnosed primary tumour (PT; n = 6), neoadjuvant chemotherapy treated tumour (CT; n = 4), and recurrent tumour (RT; n = 5). Here we have selected only those patients who have given cisplatin in their neoadjuvant chemotherapy cycle before surgery. Whereas in recurrent patient, the treatment regimen may differ (with or without chemotherapy and/or radiotherapy). Anticipating the idea, the recurrent tumours will be more aggressive or resistant to therapy compared to other two groups. Detailed patient history is provided in Supplementary Table S6. Total RNA was isolated from patient’s samples using tissue lyser (TissueLyser LT, QIAGEN) and RNeasy Plus mini kit (Qiagen). To analyse the mature miRNA expression the protocol was same as before by using miScript II RT kit (Qiagen) and miScript SYBR Green PCR kit (Qiagen). The negative expression values of a particular miRNA (−∆∆C_T_) was plotted in the box plot after normalisation to the U6 (endogenous control) expression and to the specific miRNA expression in adjacent normal tissue samples with the respective tumour sample using R software (version 3.2.1). The significance of miRNA expression level was determined by the Mann-Whitney test.

### Collection of mRNA target genes for deregulated miRNAs

Experimentally known target mRNAs of the deregulated miRNAs were collected from the miRTarBase database^22^. 42 miRNAs were found to be differentially expressed in SCC084/R and SCC131/R cell lines relative to their parental SCC084 and SCC131 lines, respectively. Fold change of their target mRNA expression in cisplatin resistant Vs sensitive scenario was collected from various multi cancer cell line data available in ONCOMINE database^21^ (Supplementary Table S12). Differentially expressed (fold change: >2.0x; *p* ≤ 0.05) mRNAs were used to create the miRNA-mRNA regulatory network using the Cytoscape software^57^. Functional categorization of the target mRNAs were performed using Gene Ontology (GO)^58^ molecular functions extracted from the Panther database^59^.

## Additional Information

**How to cite this article**: Ghosh, R. D. *et al*. MicroRNA profiling of cisplatin-resistant oral squamous cell carcinoma cell lines enriched with cancer-stem-cell-like and epithelial-mesenchymal transition-type features. *Sci. Rep.* **6**, 23932; doi: 10.1038/srep23932 (2016).

## Supplementary Material

Supplementary Information

Supplementary Table S1

Supplementary Table S2

Supplementary Table S3

Supplementary Table S4

Supplementary Table S5
