# Supplementary material for: MicroRNA profiling of cisplatin-resistant oral squamous cell carcinoma cell lines enriched with cancer-stem-cell-like and epithelial-mesenchymal transition-type features
Source: Sci Rep. 2016 Apr 5;6:23932. doi: 10.1038/srep23932 (PMC4820705; doi:10.1038/srep23932)
Supplement: Supplementary Information [file srep23932-s1.doc]

**Supplementary Information**

**MicroRNA profiling of cisplatin-resistant oral squamous cell carcinoma cell lines enriched with cancer-stem-cell-like and epithelial-mesenchymal transition-type features**

Ruma Dey Ghosh^1*^, Sangeeta Ghuwalewala^1^, Pijush Das^1^, Sapan Mandloi^2^, Sk Kayum Alam^1^, Jayanta Chakraborty^3^ Sajal Sarkar^3^, Saikat Chakrabarti^2^, Chinmoy Kumar Panda^4^, and Susanta Roychoudhury^1^

^1^Cancer Biology and Inflammatory Disorder division, ^2^Structural Biology and Bio-Informatics Division, CSIR-Indian Institute of Chemical biology, Kolkata, India. ^3^Department of Surgical Oncology, ^4^Department of Oncogene Regulation, Chittaranjan National Cancer Institute, 37, S.P. Mukherjee Road, Kolkata, India.

*Corresponding author: Ruma Dey Ghosh, PI, DST WOS-A Project, Cancer Biology and Inflammatory Disorder Division, CSIR-Indian Institute of Chemical Biology, 4, Raja S.C. Mullick Road, Kolkata-7000032, India. E-mail: [deyrumai@yahoo.co.in](mailto:deyrumai@yahoo.co.in).

**Supplementary Table S1:**

Clinico-pathological information of the UPCI: SCC cell lines* established from HNSCC tumour specimens.

|  | **UPCI: SCC131^*^** | **UPCI: SCC084^*^** |
| --- | --- | --- |
| **Origin** | New primary (73 age, Male) | Recurrant (52 age, Male) |
| **Site** | FOM (Floor of the mouth) | RMT (Retromolar trigone) |
| **Grade** | Grade-1; Well differentiated | Grade-2; Moderately differentiated |
| **Stage** | T2N2 | T2N2B |
| **11q13** | Amplification present | Amplification present |
| **p53 status** | Wild type | Wild type |

^*^ (White et al., 2007)

**Supplementary Table S2**

IC_50_ values and Resistance (R) index of cisplatin for SCC131/R and SCC084/R cells and respective parental, SCC131 and SCC084 cells in different time points.

| **IC_50_ (μM)** | | | | | | |
| --- | --- | --- | --- | --- | --- | --- |
|  | **SCC131** | **SCC131/R** | **R Index** | **SCC084** | **SCC084/R** | **R Index** |
| **24 H** | Und. | Und. | Und. | Und. | Und. | Und. |
| **48 H** | 4.74 ± 0.23 | 8.58 ± 0.3^***^ | 1.8 ± 0.01 | 9.17 ± 0.11 | 15.6 ± 1.22^***^ | 1.7 ± 0.001 |
| **72 H** | ^a^1.31 ± 0.52 | 4.62 ± 0.21^***^ | 3.5 ± 0.02 | ^a^4.69 ± 0.13^**^ | 7.85 ± 0.25^***^ | 1.6 ± 0.001 |

^a^ SCC084 cells are more resistant than SCC131 cells (*p*<0.01), ***p*<0.01, ****p*<0.001

**Supplementary Table S3**

The list of deregulated miRNAs in the tumour samples of patients with head and neck cancer compared to adjacent normal.

| **Deregulated miRNAs in HNSCC patients’-samples (PhenomiR database v2.0)^*^** | |
| --- | --- |
| **Upregulated↑** | **Downregulated↓** |
| **has-let-7i, has-mir-124-1, has-mir-128-1, has-mir-130b, has-mir-132, has-mir-134, has-mir-137, has-mir-142, has-mir-146a, has-mir-146b, has-mir-147, has-mir-154, has-mir-155, has-mir-17, has-mir-181a-1, has-mir-181b-1, has-mir-181c, has-mir-181d, has-mir-184, has-mir-18a, has-mir-18b, has-mir-193b, has-mir-197, has-mir-198, has-mir-205, has-mir-21, has-mir-221, has-mir-29c, has-mir-30a, has-mir-31, has-mir-325, has-mir-338, has-mir-34b, has-mir-34c, has-mir-372, has-mir-455, has-mir-491** | has-mir-100, has-mir-107, has-mir-125b-1, has-mir-133a-1, has-mir-133b, has-mir-138-1, has-mir-139, has-mir-149, has-mir-194-1, has-mir-195, has-mir-219-1, has-mir-26b, has-mir-375, has-mir-494, has-mir-99a |
| **Total: 37 miRNAs** | **Total: 15 miRNAs** |

^*^(As on May 2014)

**Supplementary Table S4**

**Differential miRNA expressions in SCC084/R and SCC131/R cell line relative to their parental SCC084 and SCC131 respectively (*p*< 0.05).**

| **SCC084/R Vs SCC084** | | | **SCC131/R Vs SCC131** | | |
| --- | --- | --- | --- | --- | --- |
| **miRNA Name** | ***p*-Value** | **FC** | **miRNA Name** | ***p*-Value** | **FC** |
| **miR-149** | 0.003681 | 0.040455 | **miR-130b** | 0.04516 | 0.124008 |
| **miR-184** | 0.000501 | 0.045937 | **miR-149** | 0.00204 | 0.173589 |
| **miR-137** | 0.003309 | 0.057041 | **miR-134** | 0.022277 | 0.378837 |
| **miR-193b** | 0.008818 | 0.061331 | **miR-198** | 0.019875 | 2.841101 |
| **miR-18a** | 0.00646 | 0.062098 | **miR-455** | 0.01225 | 3.158413 |
| **miR-181d** | 0.000233 | 0.066475 | **miR-147** | 0.012392 | 3.545895 |
| **miR-30a** | 0.000444 | 0.089142 | **miR-181d** | 0.006085 | 5.104077 |
| **miR-18b** | 0.000713 | 0.095643 | **miR-142-5p** | 0.022187 | 5.980019 |
| **miR-132-3p** | 0.000857 | 0.099518 | **miR-491** | 0.007642 | 9.686665 |
| **miR-31** | 0.006206 | 0.10509 | **miR-146b** | 0.000706 | 10.82916 |
| **miR-146b** | 0.005121 | 0.114352 | **miR-154** | 0.010607 | 12.76215 |
| **miR-205** | 0.001262 | 0.120054 | **miR-146a** | 0.000317 | 19.56021 |
| **miR-195** | 0.023486 | 0.133245 |  |  |  |
| **miR-494** | 0.008941 | 0.133823 |  |  |  |
| **miR-325** | 0.00055 | 0.143971 |  |  |  |
| **miR-194** | 0.010992 | 0.145486 |  |  |  |
| **miR-221** | 0.015813 | 0.167723 |  |  |  |
| **miR-125b** | 0.00063 | 0.177139 |  |  |  |
| **miR-197** | 0.024916 | 0.177286 |  |  |  |
| **miR-17** | 0.016949 | 0.182928 |  |  |  |
| **miR-26b** | 0.040602 | 0.186429 |  |  |  |
| **miR-29c** | 0.001318 | 0.186947 |  |  |  |
| **miR-134** | 0.015234 | 0.191504 |  |  |  |
| **miR-let 7i** | 0.000827 | 0.196105 |  |  |  |
| **miR-21** | 0.001004 | 0.201681 |  |  |  |
| **miR-181b** | 0.013836 | 0.2152 |  |  |  |
| **miR-155** | 0.018422 | 0.237475 |  |  |  |
| **miR-130b** | 0.004791 | 0.253077 |  |  |  |
| **miR-181a-1** | 0.028153 | 0.263286 |  |  |  |
| **miR-99a** | 0.004828 | 0.286608 |  |  |  |
| **miR-107** | 0.02407 | 0.296736 |  |  |  |
| **miR-138** | 0.007386 | 0.343135 |  |  |  |
| **miR-124-3p** | 0.022889 | 0.344554 |  |  |  |
| **miR-372** | 0.008816 | 0.346289 |  |  |  |
| **miR-338-3p** | 0.026599 | 0.571747 |  |  |  |
| **miR-491** | 0.026664 | 1.66688 |  |  |  |

**Supplementary Table S5**

**Resistance specific miRNA expressions altered in both SCC084/R and SCC131/R cell line compared to their parental SCC084 and SCC131 respectively (*p*< 0.05).**

|  | **SCC131** | | **SCC084** | |
| --- | --- | --- | --- | --- |
| **miRNA Name** | ***p*-Value** | **FC** | ***p*-Value** | **FC** |
| **miR-130b** | 0.04516 | 0.124↓ | 0.004791 | 0.253↓ |
| **miR-149** | 0.00204 | 0.174↓ | 0.003681 | 0.040↓ |
| **miR-134** | 0.022277 | 0.379↓ | 0.015234 | 0.192↓ |
| **miR-181d** | 0.006085 | 5.104↑ | 0.000233 | 0.066↓ |
| **miR-491** | 0.007642 | 9.687↑ | 0.026664 | 1.667↑ |
| **miR-146b** | 0.000706 | 10.829↑ | 0.005121 | 0.114↓ |


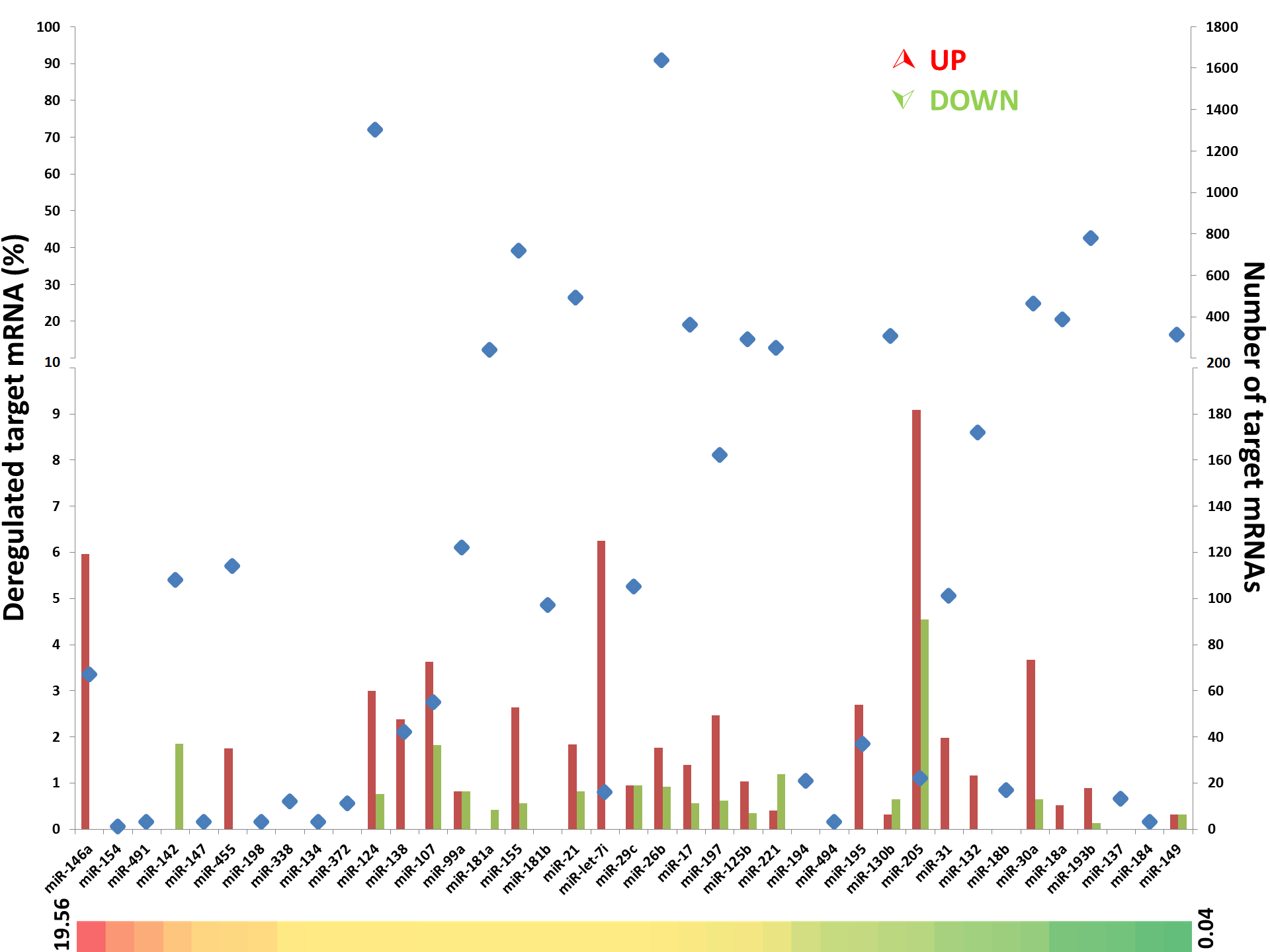


**Supplementary Figure S1**

**Deregulation of target mRNA genes with respect to the miRNA expression status in cisplatin resistant condition.** X axis represents the deregulated miRNA along with their expression fold change shown via colour bar. Y axes represent the percentage of deregulated target mRNA (as bars; RED: UP regulated and GREEN: DOWN regulated) and number of target mRNAs (as scatter; blue colour).

**
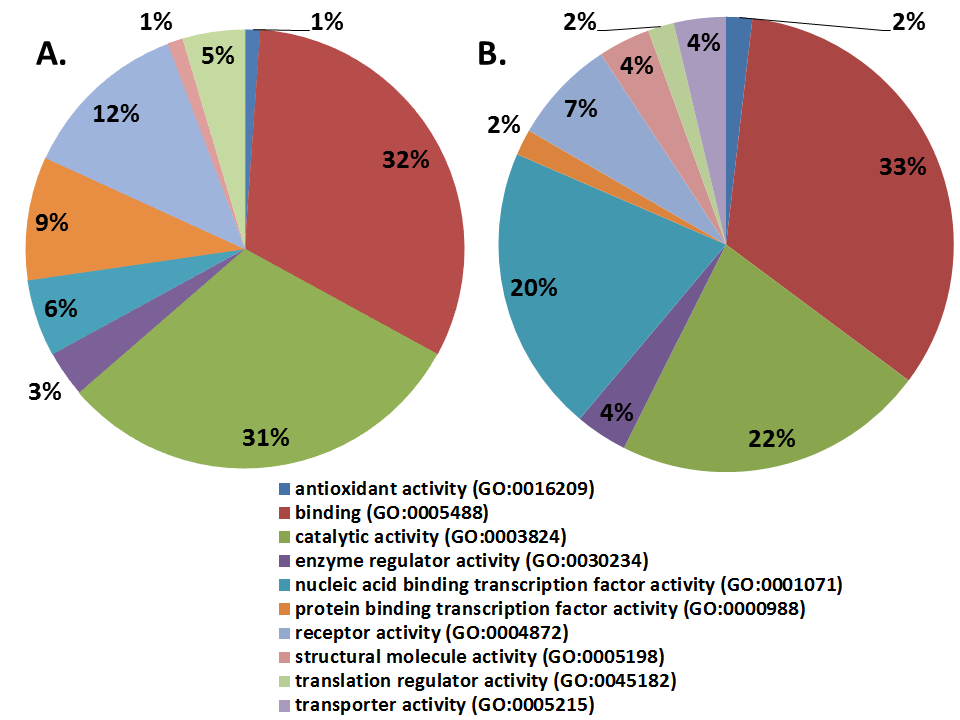
**

**Supplementary Figure S2**

**Gene Ontology (GO) functions of the deregulated mRNA target genes.** Panel A and B provide the GO functions of the upregulated and downregulated mRNA target genes, respectively.

**Supplementary Table** S10

| **Name** | **Primer Sequence 5'-3'** |
| --- | --- |
| **hsa-let-7i** | ACACTCCAGCTGGGTGAGGTAGTAGT |
| **MIR100** | ACACTCCAGCTGGGAACCCGTAGATCCGAA |
| **MIR107** | ACACTCCAGCTGGGAGCAGCATTGTACAG |
| **MIR124-3p** | ACACTCCAGCTGGGTAAGGCACGCG |
| **MIR125b** | ACACTCCAGCTGGGTCCCTGAGACCCTA |
| **MIR128-3p** | ACACTCCAGCTGGGTCACAGTGAAC |
| **MIR130b** | ACACTCCAGCTGGGCAGTGCAATGA |
| **MIR132-3p** | ACACTCCAGCTGGGTAACAGTCTAC |
| **MIR133a** | ACACTCCAGCTGGGAGCTGGTAAAA |
| **MIR133b** | ACACTCCAGCTGGGTTTGGTCCCC |
| **MIR134** | ACACTCCAGCTGGGTGTGACTGGT |
| **MIR137** | ACACTCCAGCTGGGTTATTGCTTAA |
| **MIR138** | ACACTCCAGCTGGGAGCTGGTGTT |
| **MIR139** | ACACTCCAGCTGGGTCTACAGTGC |
| **MIR142-5p** | ACACTCCAGCTGGGCATAAAGTAG |
| **MIR146a** | ACACTCCAGCTGGGTGAGAACTGAATTCCATG |
| **MIR146b-5p** | ACACTCCAGCTGGGTGAGAACTGAATTCCATA |
| **MIR147** | ACACTCCAGCTGGGGTGTGT |
| **MIR149** | ACACTCCAGCTGGGTCTGGCTCCGTGTC |
| **MIR154** | ACACTCCAGCTGGGTAGGTTATCC |
| **MIR155** | ACACTCCAGCTGGGTTAATGCTAA |
| **MIR17** | ACACTCCAGCTGGGCAAAGTGCTT |
| **MIR181a-1** | ACACTCCAGCTGGGAACATTCAACG |
| **MIR181b-1** | ACACTCCAGCTGGGAACATTCATTGC |
| **MIR181c** | ACACTCCAGCTGGGAACATTCAACC |
| **MIR181d** | ACACTCCAGCTGGGAACATTCATTGT |
| **MIR184** | ACACTCCAGCTGGGTGGACGGAGAAC |
| **MIR18a** | ACACTCCAGCTGGGTAAGGTGCATCTAGTGCAGA |
| **MIR18b** | ACACTCCAGCTGGGTAAGGTGCATCTAGTGCAGT |
| **MIR193b** | ACACTCCAGCTGGGCGGGGT |
| **MIR194** | ACACTCCAGCTGGGTGTAACAGCAACTC |
| **MIR195** | ACACTCCAGCTGGGTAGCAGCACAGA |
| **MIR197** | ACACTCCAGCTGGGTTCACCACCT |
| **MIR198** | ACACTCCAGCTGGGGGTCCAGAGGG |
| **MIR205** | ACACTCCAGCTGGGTCCTTCATTC |
| **MIR21** | ACACTCCAGCTGGGTAGCTTATCA |
| **MIR219** | ACACTCCAGCTGGGTGATTGTCCA |
| **MIR221** | ACACTCCAGCTGGGAGCTACATTG |
| **MIR26b** | ACACTCCAGCTGGGTTCAAGTAATTC |
| **MIR29c** | ACACTCCAGCTGGGTAGCACCATTTGAAATCG |
| **MIR30a** | ACACTCCAGCTGGGTGTAAACATCCTC |
| **MIR31** | ACACTCCAGCTGGGAGGCAAGATG |
| **MIR325** | ACACTCCAGCTGGGCCTAGTAGGT |
| **MIR338-3p** | ACACTCCAGCTGGGTCCAGCATCA |
| **MIR34b-3p** | ACACTCCAGCTGGGCAATCACTAA |
| **MIR34c** | ACACTCCAGCTGGGAGGCAGTGTA |
| **MIR372** | ACACTCCAGCTGGGAAAGTGCTGC |
| **MIR375** | ACACTCCAGCTGGGTTTGTTCGTT |
| **MIR455** | ACACTCCAGCTGGGGCAGTCCATG |
| **MIR491** | ACACTCCAGCTGGGAGTGGGGAAC |
| **MIR494** | ACACTCCAGCTGGGAGGTTGTCCGTG |
| **MIR99a** | ACACTCCAGCTGGGAACCCGTAGAT |
| **RNU6B** | GTGCTCGCTTCGGCAGCACATATAC |

**Supplementary Table S11**

| **Name** | **Primer Sequence 5'-3'** |
| --- | --- |
| **ABCG2** | **Forward:** CTGAGATCCTGAGCCTTTGG |
|  | **Reverse:** AAGCCATTGGTGTTTCCTTG |
| **ABCB1** | **Forward:** ACTCACTTCAGGAAGCAACCA |
|  | **Reverse:** CGGATTGACTGAATGCTGATT |
| **BIRC5** | **Forward:** GTTGCGCTTTCCTTTCTGTC |
|  | **Reverse:** TCCGCAGTTTCCTCAAATTC |
| **CTNNB1** | **Forward:** TCGAGGACGGTCGGACT |
|  | **Reverse:** ATTGTCCACGCTGGATTTTC |
| **NOTCH1** | **Forward:** GGTGAGACCTGCCTGAATG |
|  | **Reverse:** GTTGGGGTCCTGGCATC |
| **BMI1** | **Forward:** CTTTCATTGTCTTTTCCGCC |
|  | **Reverse:** TCGTTGTTCGATGCATTTCT |
| **MMP9** | **Forward:** ACGACGTCTTCCAGTACCGA |
|  | **Reverse:** GCACTGCAGGATGTCATAGG |
